# Paper Functionalized with Nanostructured TiO_2_/AgBr: Photocatalytic Degradation of 2–Propanol under Solar Light Irradiation and Antibacterial Activity

**DOI:** 10.3390/nano10030470

**Published:** 2020-03-05

**Authors:** Mouheb Sboui, Soraa Bouattour, Michelangelo Gruttadauria, Giuseppe Marcì, Leonarda Francesca Liotta, Sami Boufi

**Affiliations:** 1 Faculty of Sciences, University of Sfax, Lions Club International (LCI), Sfax BP1171-3018, Tunisia; sboui.mouheb@gmail.com (M.S.); soraa.boufi@yahoo.com (S.B.); 2 Dipartimento di Scienze e Tecnologie Biologiche, Chimiche e Farmaceutiche (STEBICEF), Università degli Studi di Palermo, Viale delle Scienze, Ed. 17, 90128 Palermo, Italy; michelangelo.gruttadauria@unipa.it; 3 Dipartimento di Ingegneria (DI), Università degli Studi di Palermo, Viale delle Scienze Ed. 6, 90128 Palermo, Italy; 4 Istituto per lo Studio dei Materiali Nanostrutturati (ISMN-CNR), Via Ugo La Malfa 153, 90146 Palermo, Italy; 5 Faculty of Sciences, University of Sfax, Laboratoire Sciences des Matériaux et Environnement, Sfax BP1171-3018, Tunisia

**Keywords:** Paper–TiO_2_–AgBr, 2-propanol photodegradation, antibacterial activity, sunlight irradiation

## Abstract

A facile method to produce paper–TiO_2_ decorated with AgBr nanoparticles by a mild hydrothermal process at 140 °C was reported. The synthesis method was based on the immersion of the paper in a ready-made suspension of TiO_2_/AgBr, comprising TiO_2_ sol solution prepared in acidic conditions and AgBr solution (10^−4^ M). A paper–TiO_2_ sample was prepared and used as reference. The formation of crystalline phases of titanium oxide (TiO_2_) and silver bromide (AgBr) was demonstrated by XRD, Raman and EDX analyses. The surface morphology of the TiO_2_–AgBr was investigated by Field Effect Scanning Electronic Microscopy (FE–SEM). The photocatalytic performances of the prepared material were evaluated in the degradation of 2-propanol in the gas phase, under simulated sunlight illumination. Its antibacterial properties against *Escherichia coli* (*E. coli)* were also assessed. The efficiency of photodegradation and the anti-bacterial properties of paper–TiO_2_–AgBr were attributed to an improvement in the absorption of visible light, the increased production of reactive oxygen species (ROS) and the low recombination of photogenerated charge carriers due to the synergistic effect between TiO_2_ and AgBr/Ag nanoparticles.

## 1. Introduction

Cellulose paper is a flexible and versatile material present in our daily lives for a wide range of applications. Besides its traditional use as the main support in printing media (e.g., newspapers, books and printing paper) and eco-friendly packaging material, cellulose-based paper arouses much interest for new innovative applications, including electronic, smart functional packaging, and sensor, energy storage and catalysis after hybridization with metallic or oxide NPs, as reported in recent interesting reviews [1,2]. The use of cellulose-based papers and fabrics as a platform in multifunctional material has attracted great attention [3,4] due to the numerous advantages offered by this class of material, including a low cost, lightweight, low thermal expansion coefficient, good thermal stability of up to 200 °C, bendability and foldability and good mechanical properties. More specifically, the hybridization of paper with TiO_2_ nanostructured coating has aroused much interest for commercial application as a deodorizing filter or indoor air purification. The first work dates back to 1995, where Matsubara et al. described the production of paper–TiO_2_ using a papermaking technique, with an effective ability to degrade acetaldehyde in air under weak fluorescent light illumination [5]. Since then, many papers were reported with a focus on improving the immobilization approach and enhancing the photocatalytic efficiency of the hybrid paper–TiO_2_ toward large classes of pollutants, including dyes, amines and nitro organic compounds, as well as volatile organic solvents. Two interesting comprehensive reviews enumerating the methods of immobilization, as well as the applications of nanoparticle-functionalized paper with different metal oxide nanostructures, including TiO_2_, have been reported by Pelton et al. [6] and Chauhan et al. [7]. The water sensitivity of paper could be addressed by a polymer additive imparting a certain hydrophobic effect, as recently reported by Garusinghe et al. [8]. They prepared water-resistant photocatalytic paper based on micro-fibrillated cellulose-polyamide-amine epichlorohydrin with immobilized TiO_2_ nanoparticles by a simple two-step mixing process, and the composite mater showed effective photocatalytic activity versus the degradation of dyes under UV-light irradiation. Examples of commercial development based on a paper–TiO_2_photocatalyst were also reported by Nippon, where newsprint coated with TiO_2_ was proposed as an effective air cleaning material under UV illumination [9]. An air-deodorizing tissue box with TiO_2_-coated paper was also proposed for air purification in the interior vehicle [9].

In our previous works, two facile methods for the preparation of paper–TiO_2_photocatalysts have been reported using either an in-situ route starting from Ti(OBu)_4_ as a precursor or a ready formed TiO_2_ sol, followed by a hydrothermal process at a low temperature [10]. It was shown that both methods produced highly efficient photocatalysts able to convert 2-propanol to carbon dioxide and water. Moreover, the composite paper–TiO_2_was revealed as competitive as TiO_2_ P25 (Evonik) powder, known to be the most effective commercial photocatalyst. Aiming to boost the photocatalytic activity under the visible light of the paper–TiO_2_ composite, Cu_2_O in the form of tiny nanoparticles (NPs) has been associated with the TiO_2_ layer to produce paper–TiO_2_–Cu_2_O and paper–Cu_2_O–TiO_2_ photocatalysts by a reduction process to form Cu_2_O nanoparticles [11]. It was shown that the inclusions of Cu_2_O in the form of tiny NPs improves photocatalytic activity and shifts the absorption edge to the visible domain. However, the paper–Cu_2_O–TiO_2_ failed to degrade organic solvents under the gas phase.

Despite the effectiveness of paper–TiO_2_ composite material of degrading organic molecules under UV-light, its widespread application in indoor cleaning air and volatile organic compounds (VOCs) treatment is handicapped by the shortcoming of TiO_2_, namely (i) the lack of a visible light response, due to its large bandgap (3.2 eV for anatase), and (ii) the rapid recombination rate of the photo-generated charge carriers (*h*^+^/*e*^−^), reducing the overall quantum yield of TiO_2_. A promising approach to alleviate these limitations consisted of coupling TiO_2_, in a controlled level, with Ag/AgBr heterojunction, giving rise to a new class of photocatalysts referred to as plasmonic photocatalysis. The presence of tiny Ag NPs generated in-situ by light reduction will extend the response of the TiO_2_ catalyst to visible light through the localized surface plasmon resonance (SPR) of noble metal nanostructures [12]. In addition, metal Ag has been found to improve the charge separation by acting as an electron trap [13]. The first reports on the use of silver halides in combination with TiO_2_ as visible-light plasmonic photocatalysts are quite recent, dating back to 2006 with the pioneering works of Hu et al. [12] (TiO_2_/Ag/AgBr) and Wang et al. [14].

Given the lack of literature data concerning the coupling of AgBr with paper–TiO_2_ composites, we considered it worthy to investigate how the coupling of AgBr with TiO_2_ would affect the efficiency of photoactive TiO_2_−containing paper for the degradation of 2-propanol as a VOC model molecule under simulated sunlight irradiation and room temperature. Due to the sensitivity of cellulose paper to thermal degradation, a mild approach for the functionalization of paper with TiO_2_–AgBr nanostructured coating was adopted. The prepared paper–TiO_2_–AgBr was characterized by Raman, XRD, UV-Vis absorption and SEM observation. This work is a continuation of our work carried out on the functionalization of paper with TiO_2_ for the degradation of VOCs [10]. Presently, the main objective is to enhance the photocatalytic activity under sunlight, taking advantage of the plasmon of Ag to transfer the energy to TiO_2_, and the property of Ag to reduce the recombination rate of the electron-hole charge carriers.

In fact, 2-propanol (C_3_H_7_OH) was chosen as a VOC model molecule in this work due to its extensive use, particularly in medical centers. It is an organic compound that belongs to the group of alcohols. 2-propanol is known for several properties, such as being volatile and flammable. It has a distinctive, non-repulsive smell, which it can be stinging when inhaling it in large doses. 2-propanol is usually used in hospitals or medical clinics, as it is often used in the installation of sterilizers (antiseptic or antibacterial). In addition, it is used in cosmetics and pharmaceutical preparations. As a result of the serious health risks connected with the existence of 2-propanol in the environment, particularly in the air (gas phase), we believe its use as a model for VOCs is interesting to study.

## 2. Materials and Methods

### 2.1. Materials

All chemical products used in this work are provided by Aldrich (Grenoble, France), including: titanium (IV) butoxide (97%); ethanol (≥99.0%); nitric acid (≥65%); potassium bromide (≥99%); silver nitrate (≥99.8%); and 2-propanol (≥99.5%). The paper used is a conventional filter paper provided by Whatman (Grenoble, France), with a grammage and thickness of 87 g/m^2^ and 180 µm, respectively.

### 2.2. Synthesis of Paper–TiO_2_–AgBr

TiO_2_ sol was synthesized as follows: 1 (*w*/*v*) % of titanium (IV) butoxide was added as a precursor to a mixture of ethanol and acetic acid (90/10 wt %) under constant stirring overnight at an ambient temperature. Subsequently, 20 mL of nitric acid solution (1 M) was added dropwise to the mixture with continuous stirring. After that, the entire mixture was placed in an autoclave and heated at 140 °C for 3 h in order to form TiO_2_ sol nanoparticles (hydrothermal procedure). An aqueous solution of AgBr was freshly prepared by mixing a solution of AgNO_3_ (10^−4^ M) with a solution of KBr (10^−4^ M), and then 20 mL of such AgBr solution (10^−4^ M) was added to TiO_2_ sol under constant stirring for 1 h at room temperature, and finally the cellulose paper was immersed in the TiO_2_–AgBr suspension for 2 h at an ambient temperature. After removing the paper from the TiO_2_–AgBr suspension, we dried it in the oven at 60 °C for 3 h. This sample is referred to as paper–TiO_2_–AgBr.

For paper–TiO_2_, the same steps were used to prepare paper–TiO_2_–AgBr, but without adding the AgBr solution.

### 2.3. Characterization

In this work, the prepared samples were characterized by several techniques: 

Raman spectra were recorded on a LabRAM Analytical Raman micro-spectrograph (Jobin-Yvon, Horiba group, Longjumeau Cedex, France) using a He–Ne laser source as exciting radiation (*λ* = 632.8 nm) and an air-cooled CCD (Charge-Coupled Device) detector. The acquisition time was 100 s.

FE–SEM characterization was performed using a Merlin FEG SEM from Carl Zeiss (ZEISS SUPRA40, Oberkochen, Germany), coupled with an SDD XMAX Energy Dispersive X-ray spectrometer (EDX) from Oxford Instruments and powered with the AZtec system (Carrollton, TX, USA). Prior to SEM analysis, the samples were coated with about 1 nm of platinum. 

X-ray diffraction patterns were registered using a BRUKER AXS diffractometer (Madison, WI, USA) with a Cu-K*α* radiation, generated at 30 kV, and an incident current of 100 mA. Samples in the form of thin film were analyzed from 2*θ* = 5° to 70°, using scanning steps of 0.05° and a time per step of 100 s.

The optical properties of the prepared sample were evaluated using a Perkin Elmer Lambda 35 spectrophotometer (Perkin Elmer, Waltham, MA, USA) operating system of between 200 and 700 nm. The UV-vis spectra were recorded in the reflectance mode on the dried samples. The reflectance *R* was obtained from each sample in the UV-vis spectral regions, and the remission function *F*(*R*), corresponding to the absorbance of the sample, was calculated using the Kubelka–Munk equation for optically thick samples.

To investigate the quantity of TiO_2_ and AgBr deposited on the surface of the paper, thermogravimetric analysis was performed using a TGA 400 (Perkin Elmer, Waltham, MA, USA). The paper sample was heated from 30 °C to 600 °C at a rate of 10 °C min^−1^ under an air flow (30 mL/min).

### 2.4. Photoactivity Experiments

All photodegradation tests of 2-propanol in the gaseous phase were carried out in a batch type reactor by prepared photocatalysts. The reactor used in this work was made of Pyrex glass, and has a cylindrical form (height: 14.0 cm, diameter: 9.0 cm and volume of 890 cm^3^).

Before starting the photodegradation process of 2-propanol, the photoreactor was purified from any gas by saturating the reactor with oxygen for 30 min. Subsequently, 2-propanol was injected by a septum positioned in a side wall of the photoreactor. The initial concentration of 2-propanol in the gas phase was adjusted to 91 µM.

Before illumination, the system is kept in dark conditions for 1 h in order to reach adsorption equilibration between the catalyst and the contaminant (2-propanol). The reactor was located inside the SOLARBOX apparatus (CO.FO.ME.GRA., Milan, Italy) and equipped from the top with a Xenon lamp (1500 W) that simulates solar power. It is also equipped with a water filter placed between the reactor and the lamp in order to cutoff the infrared radiation and maintain the temperature inside the reactor at 27 °C. In this work, the ultraviolet irradiation emitted by the Xenon lamp used was equal to 10 W m^−2^ in the range of 315–400 nm, and was measured by the UVX Digital radiometer. The photocatalytic test for each sample prepared in this work lasted about 5 h of illumination. During fixed time periods, 200 µL of gas was withdrawn from the photoreactor by means of a gas-tight syringe for analysis.

Concentrations of 2-propanol and intermediate products were monitored by a GC–17A Shimadzu gas chromatograph equipped with a HP–1 column and a flame ionization detector (FID) (Shimadzu Europa GmbH, Albert-Hahn-Str. 6-10, Duisburg, Germany), while CO_2_ was measured by a 60/80 Carboxen column in an HP6890 gas chromatograph equipped with a TCD (Thermal Conductivity Detector).

### 2.5. Bacteria Preparation and Antibacterial Activity Tests

The antibacterial activity was evaluated by the disc diffusion method, without the evaluation of possible silver released, using *E. coli* ATCC 25,922 as (*Gram-*) bacterium [15]. In brief, the bacterial preinoculum cultures were grown overnight at 37 °C in 20 mL of nutrient broth (made of 1 g/L beef extract; 5 g/L neutralized peptone; 2 g/L yeast extract; and 5 g/L NaCl) subjected to horizontal shaking at 250 rpm. Then, 100 µL of the culture suspension with a concentration of 10^6^ CFU/mL was spread on the agar plates. Circular samples of paper–TiO_2_–AgBr and of the neat paper (used for comparison) were cut (approximately 1.5 cm in diameter) and placed on the surface of the inoculated culture medium. After incubation under daylight for 24 h at 37 °C, samples were visually examined for the growth of bacteria in the area surrounding the film.

## 3. Results and Discussion

### 3.1. Raman Spectroscopy

**Figure 1:**
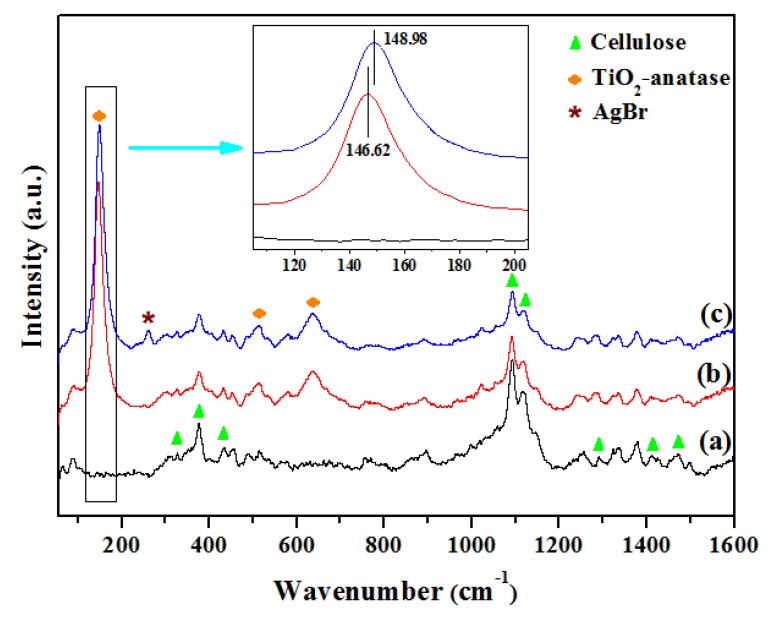
Raman spectra of neat paper (**a**), paper–TiO_2_ (**b**) and paper–TiO_2_–AgBr (**c**).

Raman was used to confirm the presence of TiO_2_ on the surface of the modified paper. Thanks to the strong Raman scattering of TiO_2_, it is possible to probe its presence, even at content lower than 1%. In Figure 1, the appearance of an intense band at ~147 cm^−1^ (*E*_g_) and two minor bands at 637 cm^−1^ (*E*_g_) and 516 cm^−1^ (A_1g_) proved the presence of crystalline TiO_2_anatase on cellulose paper [10,16]. According to the literature, the band at ~147 cm^−1^ (*E*_g_) is assigned to the symmetric stretching vibration of O–Ti–O, while the two bands at 637 cm^−1^ (*E*_g_) and 516 cm^−1^ (A_1g_) are due, respectively, to the symmetric stretching vibration and antisymmetric bending vibration of O–Ti–O in TiO_2_. Features of cellulose were also observed, with typical bands at 329, 375, 439, 1094, 1116, 1292, 1414 and 1469 cm^−1^, which are consistent with cellulose I data [10,17]. No evolution of the frequency of the cellulose bands was noted following the functionalization process, suggesting that the grafting of TiO_2_ took place at the surface of the cellulose fiber, and also that the cellulose paper continued to maintain its crystal structure.

For paper–TiO_2_–AgBr, the intensities of the three characteristic peaks of anatase TiO_2_ changed compared to those observed for paper–TiO_2_, and such an effect might be induced by the presence of AgBr [18,19,20]. The greatest peak at 146.62 cm^−1^ detected for the paper–TiO_2_ shifted at higher wavenumbers of 148.98 cm^−1^ after loading the AgBr. In addition, the peak at 259 cm^−1^, which is typical of AgBr, was clearly detected, along with both bands of TiO_2_ and cellulose, respectively, confirming the successful loading of AgBr and TiO_2_ on the surface of the paper.

### 3.2. SEM Observation

**Figure 2:**
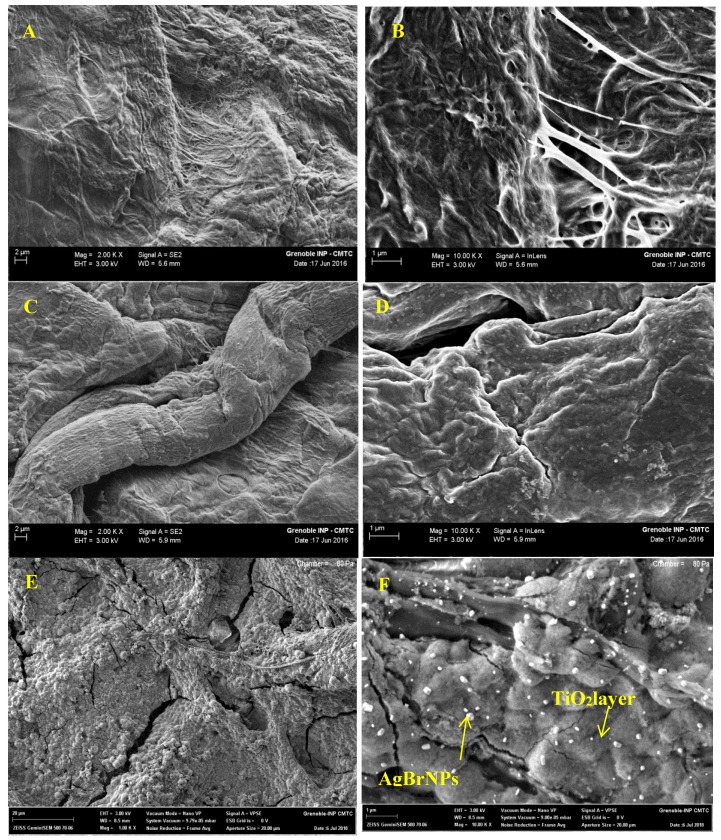
FE–SEM images of neat paper (**A**,**B**), paper–TiO_2_ (**C**,**D**) and paper–TiO_2_–AgBr (**E**,**F**).

**Figure 3:**
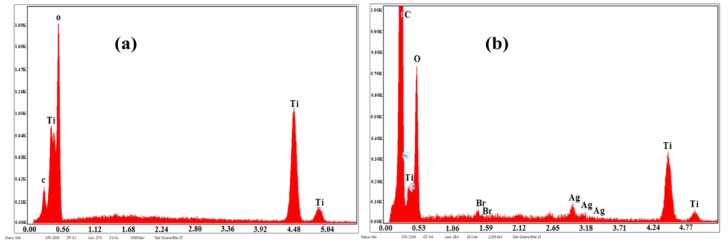
EDX spectra of paper–TiO_2_ (**a**) and paper–TiO_2_–AgBr (**b**).

In order to study the surface morphology of various prepared samples, the FE–SEM technique was adopted. The FE–SEM images of paper cellulose before and after being treated with TiO_2_ are shown in Figure 2. The untreated paper comprises an intertwined fibrous network, with the regulation of fibers in a random plane orientation, which is the origin of the strength of paper. Regarding paper treated with TiO_2_, fine elements of cellulose fibers no longer appeared, masked by a thin coating layer, presumably associated to TiO_2_. In paper–TiO_2_–AgBr, the surface of the paper is coated with a layer of TiO_2_, masking the morphological details of cellulose fibers, revealing at higher magnification tiny NPs of about 50 to 100 nm in size immobilized on the top of the TiO_2_ layer. Further indication about the chemical composition of the layer coating the surface of the paper was provided from EDX analysis (Figure 3), revealing the presence of Ti, or Ti, Ag and Br, in addition to C and O, in paper–TiO_2_ and paper–TiO_2_–AgBr, respectively.

### 3.3. XRD Characterization

**Figure 4:**
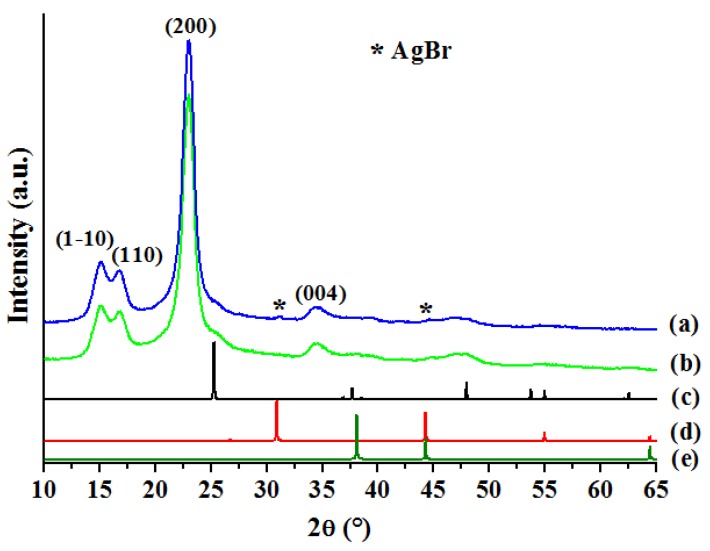
X-ray diffractograms of fresh paper–TiO_2_–AgBr (**a**), paper–TiO_2_–AgBr after photocatalytic test (**b**), TiO_2_ anatase ICSD reference (*n*° 9853) (**c**), AgBr ICSD reference (*n*° 52246) (**d**), metallic Ag ICSD reference (*n*° 44387) (**e**).

The XRD pattern of paper–TiO_2_–AgBr is shown in Figure 4, with the superposition of the diffraction pattern of AgBr and TiO_2_anatase. The main intensity peaks that are clearly visible are those corresponding to cellulose I at 14.9°, 16.6°, 22.65°and 34.4° 2*θ* assigned to (1–10), (110), (200) and (004), respectively [21]. Other minor peaks with weak intensity at 30.9°, 44.3° and 55.0° 2*θ* were assigned to the AgBr phase, according to the Inorganic Crystal Structure Database (ICSD) reference. The diffraction peaks of TiO_2_ anatase—the presence of which was confirmed by Raman (Figure 1)—cannot be clearly identified, although an evidentshoulder in correspondence of the most intense peak of the TiO_2_ anatase ICSD reference at 25.2° 2*θ* can be seen in the diffraction patterns of both samples of the paper–TiO_2_–AgBr (as prepared and after the photocatalytic test, curves a, b). 

After the photocatalytic test, the peaks assigned to AgBr (marched with asterisk *) disappeared, likely due to the formation of metallic Ag not easily detectable due to its nanometric size, and/or because the mean peak at ~38.2° 2*θ* can be overlapped by secondary TiO_2_ anatase features.

### 3.4. UV-Vis Characterization

**Figure 5:**
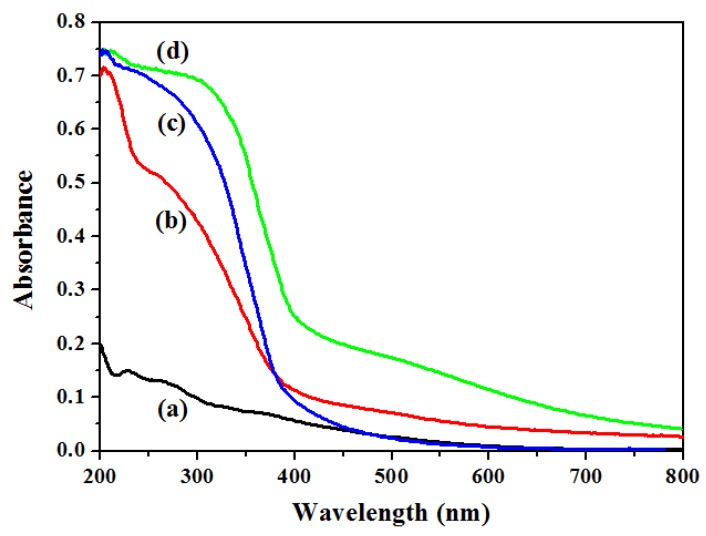
UV–vis absorption spectra of neat paper (**a**), paper–TiO_2_ (**b**), fresh paper–TiO_2_–AgBr (**c**) and paper–TiO_2_–AgBr after photocatalytic test (**d**).

The UV-Vis absorption spectra of neat paper, paper–TiO_2_, fresh paper–TiO_2_–AgBr and paper–TiO_2_–AgBr after the photocatalytic test under simulated sunlight illumination are shown in Figure 5. The neat paper presents a weak absorption in the UV domain without any absorption in the visible range, which is consistent with the chemical structure of paper composed mainly of cellulose. The weak absorption in the UV is presumably due to the presence of small residual lignin in cellulose fibers. In paper–TiO_2_, absorption in the UV-domain of between 300 to 400 nm was strongly enhanced, which is consistent with the optical properties of TiO_2_ with a bandgap of around 3.2 eV. In paper–TiO_2_–AgBr stored out of light, the absorption in the UV domain is further enhanced in comparison with that of paper–TiO_2_, without any absorption in the visible domain. After exposure to sunlight stimulant and photocatalytic degradation test, the absorption in the UV-domain further increased, and a broad absorption in the visible range of between 450–600 nm can be seen. This absorption could be due to the formation of Ag NPs on the surface of immobilized AgBr particles by the Ag^+^ photoreduction under sunlight irradiation. Indeed, this absorption band, in the visible range, corresponds to the Ag surface plasmon resonance (SPR). 

### 3.5. Thermogravimetric Analysis (TGA)

**Figure 6:**
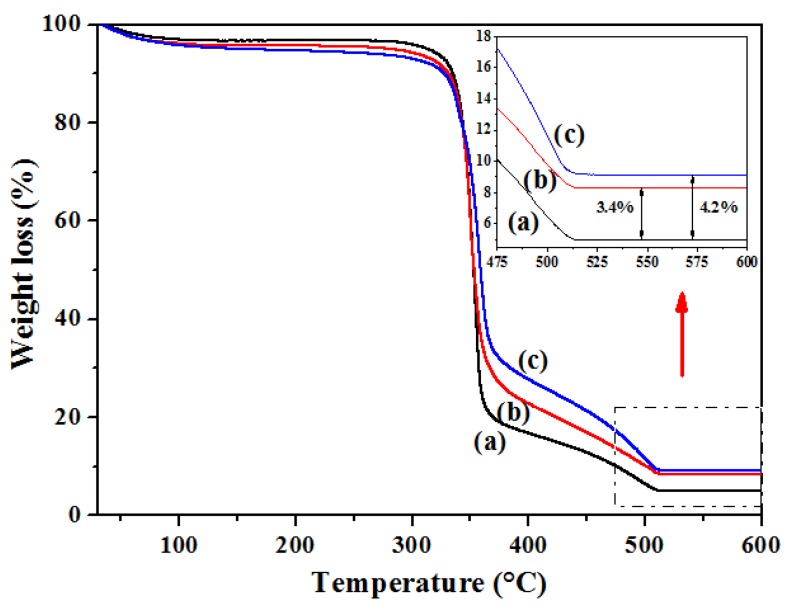
Thermogravimetric analysis curves of neat paper (**a**), paper–TiO_2_ (**b**) and paper–TiO_2_–AgBr (**c**).

Under air flow, TGA analysis of the different paper samples has been performed to assess the thermal stability after surface functionalization, and also estimate the amount of TiO_2_–AgBr appended on paper (Figure 6). For all studied samples, the material was stable up to 250 °C, followed by a rapid weight loss from 280 to 350 °C, related to the deterioration of the cellulose structure through the cleavage of the glycosidic bonds. No meaningful evolution of the initial temperature of thermal degradation could be seen after the modification with TiO_2_ and TiO_2_–AgBr, meaning that the presence of TiO_2_ and AgBr did not improve the thermal stability of the paper substrate. The ash content for neat paper, paper–TiO_2_ and paper–TiO_2_–AgBr was equal to 4.9, 8.3 and 9.1 wt%, respectively. This means that the amounts of TiO_2_ or TiO_2_ and AgBr present in the loaded paper were about 3.4% and 4.2% for paper–TiO_2_ and paper–TiO_2_–AgBr, respectively. 

### 3.6. Photocatalytic Activity

**Figure 7:**
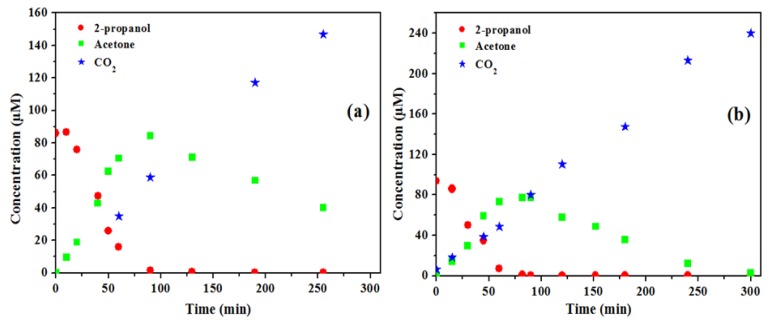
Photodegradation of 2-propanol and formation of acetone and CO_2_ versus irradiation time in the presence of paper–TiO_2_ (**a**) and paper–TiO_2_–AgBr (**b**).

In order to check the efficiency of prepared photocatalysts, they were used to remove 2-propanol, as a model of VOCs, under simulated sunlight illumination and in dry conditions. Figure 7 displays the photodegradation of 2-propanol and formed the intermediate products as a result of photocatalytic activity of both paper–TiO_2_ and paper–TiO_2_–AgBr.

Before illumination, the system was kept in dark conditions for 1 h in order to reach the adsorption equilibration between the catalyst and 2-propanol, although the adsorption of 2-propanol was always negligible. 

In order to check the effect of light, oxygen and catalysts in the photodegradation process of 2-propanol, some blank reactivity tests of 2-propanol in the gas phase were conducted under the same experimental conditions, but without light, oxygen or a catalyst. All these tests concluded that there is no reactivity, which means that the presence of light, oxygen and a catalyst are necessary factors for the photocatalytic process. Moreover, in order to investigate the stability of the cellulose paper that we adopted in our work as a support, two blank tests were conducted for both untreated paper and paper treated with TiO_2_ (paper–TiO_2_) in the same experimental conditions as before, but in the absence of 2-propanol and for 12 h of illumination. These two tests confirmed the stability of the paper under illumination. 

Figure 7 shows the ability of photocatalysts prepared for the complete degradation of 2-propanol and the formation of acetone and carbon dioxide as intermediate products for this photodegradation. We can also note that when the concentration of 2-propanol decreases and reaches a minimal value, then the degradation of acetone to carbon dioxide is started. However, the degradation of 2-propanol was faster when AgBr nanoparticles were associated with a TiO_2_ layer in the case of paper–TiO_2_–AgBr as a photocatalyst, confirming the effective and important role of the AgBr/Ag NPs in the photodegradation process. To compare the activity of the different samples, the initial reaction rate of 2-propanol was calculated in the presence of both catalysts, and the results are reported in Table 1. This table confirms the important and useful role of AgBr NPs in improving the photodegradation process of 2-propanol under sunlight. The increase of the initial reaction rate in the presence of paper–TiO_2_–AgBr was not so much impressive, but it is important to underline that only in the presence of paper–TiO_2_–AgBr, as a catalyst, the acetone, formed during the 2-proponol degradation, was completely degraded during the 5 h of irradiation. On the contrary, in the presence of the other catalyst, without AgBr, only a partial degradation of acetone was reached after the 5 h of irradiation, indicating one more time the best performances of the paper–TiO_2_–AgBr catalyst. The reasons for the increased efficiency of paper–TiO_2_–AgBr with respect to paper–TiO_2_ can be attributed to an improvement in the absorption of visible light (see Figure 5), which should increase the production of reactive oxygen species (ROS). Moreover, thanks to the synergistic effect between TiO_2_ and AgBr/Ag nanoparticles, it could be also hypothesized that there is a lower recombination rate of photogenerated charge carriers [22]. In Figure 7, it also reported the evolution, in the gas phase, of the amount of CO_2_ deriving from the mineralization of 2-propanol. For both catalysts, the carbon balance was fulfilled, indicating that no other carbon containing species were formed, but 2-propanol was totally mineralized only in the presence of paper–TiO_2_–AgBr as catalyst, at least after 5 h of irradiation. This fact is very important for a possible use of these materials for environmental remediation, as it indicates that paper–TiO_2_–AgBr is able not only to degrade the substrate, but also mineralize it to innocuous species.

### 3.7. Mechanism of Transfer and Separation of Photogenerated Charges

**Scheme 1:**
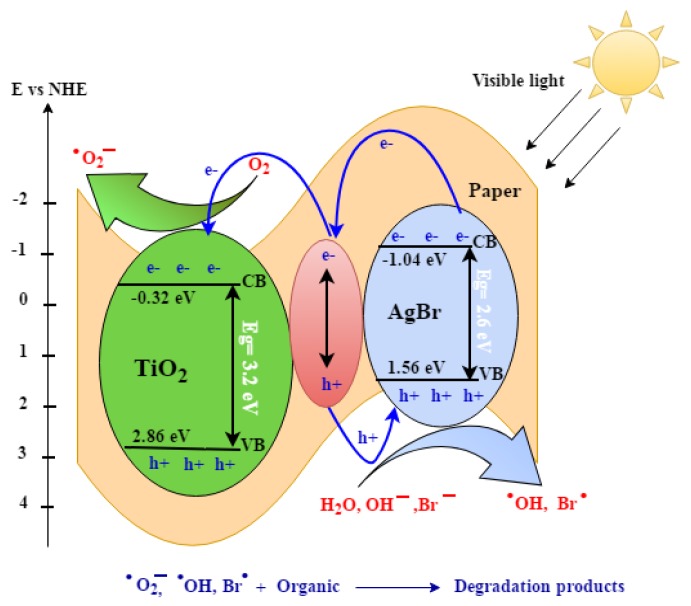
Photocatalytic mechanism scheme of paper–TiO_2_–AgBr under sunlight irradiation.

The major photocatalytic reaction steps involved in the photocatalytic degradation of 2-propanol in the presence of paper–TiO_2_–AgBr can be described as follows: under sunlight illumination, AgBr in contact with the TiO_2_ layer would form by the photoreductionof Ag^0^ cluster species deposited on the surface of AgBr. Indeed, some evidences of Ag^0^ formation can be deduced from the appearance under sunlight irradiation of a broad absorption band in the visible range of between 450–600 nm (see Section 3.4). However, additional work is needed to confirm the presence of Ag^0^ species by XPS experiments. The Ag/AgBr can be excited by visible light generating photoinduced electrons (*e*^−^) and holes (*h*^+^). The *e*^−^ will quickly transfer to TiO_2_ via the heterojunction, while *h*^+^ moves to AgBr. The electrons are captured by adsorbed oxygen forming superoxide radicals ^•^O_2_^−^, while the holes combine with H_2_O, OH^−^/or Br^−^ ions to form ^•^OH/or Br^•^ radicals. All of ^•^O_2_^−^, ^•^OH and Br^•^ were reactive species capable of the degradation of organic molecules. Scheme 1 illustrates the hypothesized mechanism. In addition, Ag^0^ is expected to reduce the recombination of photogenerated charge carriers, as reported in literature data concerning TiO_2_/Ag/AgBr composites [12]. 

### 3.8. Antibacterial Properties

**Figure 8:**
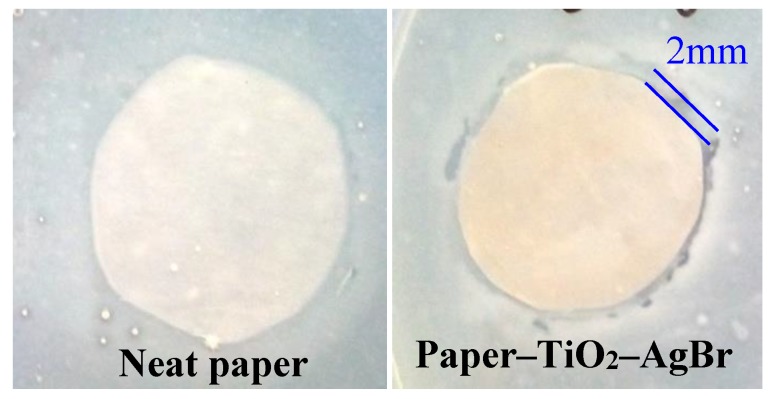
Digital images of the neat paper and paper–TiO_2_–AgBr samples placed in *E. coli* culture medium after incubation at 37 °C for 24 h under daylight.

The antibacterial properties of paper–TiO_2_–AgBr were qualitatively assessed by disk-diffusion protocol using *E. coli* as *Gram*–bacteria to evaluate the aptitude of modified paper to resist bacteria growth. For comparison reasons, the experiment was repeated by using neat paper. In material lacking an antibacterial effect, bacteria would adhere to the surface of the films and proliferate around the sample, while an inhibition hallow around the films would be observed for samples endowed with a bactericidal effect. In Figure 8, the digital images of the neat paper and paper–TiO_2_–AgBr placed in an inoculated culture medium of *E. coli* are compared after incubation at 37 °C for 24 h under daylight illumination.

The neat paper did not show any antibacterial activity, which is expected due to the sensitivity of cellulose to bacterial attacks. By contrast, the sample paper–TiO_2_–AgBr showed a clear inhibition zone at the outer edge, indicating that bacteria cannot proliferate nearby the sample. The activity of paper–TiO_2_–AgBr is presumably due to the presence of Ag/AgBr coupled with TiO_2_, which is able to kill bacteria after exposure to visible light through a combination of a multitude of effects [23]: (i) the interaction of Ag with the bacteria outer membrane, inducing structural changes that lead to cell death; (ii) the interaction of released silver ions with sulfur and phosphorus in DNA, resulting in the inactivation of the DNA replication; and (iii) the generation of reactive oxygen species (ROS) upon exposure to visible light, catalyzing the destruction of bacteria through a photocatalytic effect [24]. The antibacterial property of paper–TiO_2_–AgBr is of great benefit for the durability of paper-based photocatalysts when their long-term use is considered. Cellulose-based substrates are known to be sensitive to bacteria proliferation [25], namely in humid atmospheres, causing the disintegration of paper through a biodegradation process. Hence, in addition to its beneficial effect on photocatalytic efficiency under simulated sunlight, the presence of AgBr will extend the shelf life of a paper–TiO_2_–AgBrphotocatalyst when exposed to a humid atmosphere.

## 4. Conclusions

In summary, a simple method to produce paper–TiO_2_ decorated with AgBr nanoparticles was reported. Using an inexpensive, non-toxic hydrothermal procedure, the TiO_2_–AgBr composite grows directly on the surface of the paper at a mild temperature (140 °C). From the FE–SEM study, it was observed that TiO_2_ formed a thin layer coating the paper surface, on the top of which AgBr was immobilized in the form of dispersed NPs in the case of paper–TiO_2_–AgBr. The Raman technique confirmed the presence of a thin layer of TiO_2_anatase on the paper surface. Paper–TiO_2_–AgBr showed good efficiency in the removal of 2-propanol in the gas phase under simulated sunlight illumination, suggesting its potential use for environmental remediation in treating polluted air. In addition, paper–TiO_2_–AgBr is characterized by a very good antibacterial ability. The enhanced photocatalytic activity was attributed to several concomitant factors, such as the good dispersion of AgBr nanoparticles, the in-situ formation of Ag^0^ NPs on the AgBr surface with enhanced visible-light absorption and the hypothesized efficient separation of the photo-generated charge (*h*^+^/*e*^−^).

In conclusion, the immobilization of TiO_2_–AgBr on a flexible support as cellulose paper offers wide possibilities for its application in the removal of indoor VOCs and bacteria. Work is in progress to investigate the photocatalytic activity under halogen lamps and LEDs.

## Figures and Tables

**Table 1:** 2-Propanol initial reaction rate calculated for the different photocatalysts was tested.

Photocatalysts	Initial Reaction Rate (mol. s^−1^)
paper−TiO_2_	1.7 × 10^−8^
paper−TiO_2_−AgBr	2.1 × 10^−8^
